# Herbal Mouthwash Containing Extracts of *Baccharis dracunculifolia* as Agent for the Control of Biofilm: Clinical Evaluation in Humans

**DOI:** 10.1155/2015/712683

**Published:** 2015-03-22

**Authors:** Vinícius Pedrazzi, Mateus Freire Leite, Reisla Cristina Tavares, Sandra Sato, Glauce Crivelaro do Nascimento, João Paulo Mardegan Issa

**Affiliations:** ^1^Department of Dental Materials and Prosthodontics, Ribeirão Preto Dentistry Faculty, University of São Paulo, 14040-904 Ribeirão Preto, SP, Brazil; ^2^Multidisciplinary Institute for Health, Federal University of Bahia, Anísio Teixeira Campus, 45029-094 Vitoria da Conquista, BA, Brazil; ^3^Department of Morphology, Physiology and Basic Pathology, Ribeirão Preto Dentistry Faculty, University of São Paulo, 14040-904 Ribeirão Preto, SP, Brazil

## Abstract

*Baccharis dracunculifolia* DC (Asteraceae), popularly known as “alecrim-do-campo,” is largely distributed in South America, is shown to exhibit protective actions against gastric ulcers, has anti-inflammatory properties, and is hepatoprotective. Several essential oils obtained from *Baccharis* species possess biological activities, such as antimicrobial and antivirus activities. This randomized controlled trial evaluated the efficacy of *B. dracunculifolia* in the reduction of dental biofilm, comparing this natural product with other mouthwashes already known in the dental market. In measuring the time after use of mouthwash (*t* = 1), there was no difference between products (*P* = 0.602); that is, subjects in the study had a similar PI after the first use. After one week (*t* = 2), there was no difference between the four products evaluated (*P* = 0.674), so, all research individuals completed the study with a similar reduction in dental biofilm between themselves but it was different from initial state (Friedman test). It is possible to conclude that *B. dracunculifolia* had the same efficiency of the materials used to oral hygiene in reduction of dental plaque and, consequently, prevention of dental caries. Thus, we can consider *B. dracunculifolia* as a good candidate for new material to be implemented in dental care.

## 1. Introduction

Dental plaque is a complex biofilm that accumulates on the surface of the teeth, containing more than 500 bacterial species. The dental plaque is produced by initial colonizing bacteria in the salivary film of the enamel, followed by secondary colonization through the interbacterial adhesion [1]. Oral infectious diseases, such as periodontal inflammation, caries, and gingivitis, can be developed by dental biofilm formation [2]. These kinds of dental problems can cause other serious health diseases and, because of this, the study in the development of referred pathologies has been a theme of growing interest [3]. In this way, recent research suggests that periodontal diseases may contribute to the development of heart diseases [4] and stroke [5] and they can compromise the health of patients with diabetes, respiratory diseases, or osteoporosis [6]. Among oral infectious diseases, caries has been the most persistent infection in the history of humanity [3], also being the leading cause of tooth loss in children and young adults [1]. Caries disease is an irreversible microbial pathology of the calcified tissues of the teeth and it is a multifactorial disorder, greatly influenced by the diet of the individual, which often leads to cavitation, since it is characterized by destruction of the organic substance of the tooth and demineralization of the inorganic portion [7, 8]. Previous literature has shown data that the mutans group of Gram-positive bacteria, particularly* Streptococcus mutans*, occupies a substantial proportion of the microbiota that integrates the cariogenic biofilm, and their participation in the etiology of dental caries is very important [9]. Its virulence is directly related to the ability to produce acids for metabolizing carbohydrates. These acids reduce the pH of the biofilm and cause demineralization of tooth structure [10].

The ability of* S. mutans* to initiate a decay depends on the association of several virulence factors, including initial adhesion to the tooth surface by adhesion glycoproteins with the ability to synthesize extracellular polysaccharides insoluble (PEC). PEC is mainly produced by means of enzymes glucosyltransferases (GTFs), which promotes the accumulation and retention of microorganisms on tooth surfaces, with high capacity to catabolize carbohydrates and produce acids that soften tooth enamel and also with ability to grow and to continue metabolism of carbohydrates at low pH [11].

Biofilm bacteria have increased antimicrobial resistance [12] and, for this reason, the increase in demand for new antimicrobials has led to several investigations toward the search for antimicrobial effects of phytochemicals extracted from a range of species of botanical origin [13, 14]. In fact, recently, much attention has been given to natural products with health-promoting benefits [15, 16].

In the process of developing new pharmacologically active compounds from natural products for using in dentistry, the Southeastern Brazilian propolis may prevent dental caries [17, 18].* B. dracunculifolia* is the most important botanical source of Southeastern Brazilian propolis, which due to its colour is called green propolis, whose healthy benefits, including the hepatoprotective effect, are well described in the literature [19]. Propolis is the generic name for the resinous substance produced by honeybees (*Apis mellifera*) and is commonly used to improve health and to prevent several diseases [20]. It has been used for medicinal purposes since ancient times, and its antimicrobial, antitumoural, and immunomodulatory activities have been reported [21].

Approximately 500 species of* Baccharis* are known, which are distributed specifically in America [22]. Among others, their potential antirheumatic, antifungal [23], antioxidant [22], and insecticide properties are known.* Baccharis* species have been extensively used in folk medicine for the treatment and prevention of anaemia, inflammation, diabetes, and stomach, liver, and prostate diseases [24].* Baccharis dracunculifolia* DC (Asteraceae), popularly known as “alecrim-do-campo,” is largely distributed in South America and is shown to exhibit protective actions against gastric ulcers [25], has anti-inflammatory properties [26], and is hepatoprotective [27]. Several essential oils obtained from* Baccharis* species possess biological activities, such as antimicrobial [28] and antivirus [29] activities.* B. dracunculifolia* and Brazilian green propolis have been reported to show similar anticariogenic [30], antiulcer [31], and immunomodulatory activities [32].

Microemulsions are systems with good thermodynamic stability and high solubilization capacity of hydrophilic and lipophilic drugs, are easy to use, and give greater stability to the most active embodied components. A microemulsion was used for extract of* B. dracunculifolia* in the present work, and its toxicity has been evaluated in our laboratory, with results of no toxicity at concentrations of use. However, there are relatively few studies reporting topical administration of microemulsions [33–35].

Considering the search for new therapeutic mouthwashes and the benefits of natural care products for general and oral health, the aim of this study was to evaluate the efficacy of* B. dracunculifolia* in the reduction of dental biofilm. In this sense, there is the possibility of finding a new type of material for use in oral hygiene that may contribute to prevention of dental caries. Thus, this study used a clinical evaluation of a pharmaceutical oral formulation based on microemulsion containing extract and essential oil of* B. dracunculifolia*, in order to propose the application of this plant material in formulations for dental use.

## 2. Materials and Methods

The present work was randomized and controlled, crossover type, in order to establish a triple-blind study (the handler of the formula, the packager/labeller, and clinical evaluation). Twelve healthy subjects, aged between 18 and 30 years, were recruited and chosen according to the following inclusion/exclusion criteria: good general health; no sign of destructive periodontal disease; minimum of 24 teeth, six in each quadrant; absence of antibiotic therapy for a period of three months before the study; no smoking; no regular use of mouthwashes.

All patients received verbal and written statement about the study in question and signed a consent form (Human Research Ethics Committee 2008.1.1061.58.4; CAAE number 0065.0.138.000-08). Twelve individuals were divided into four groups that received rinses solutions each (Table 1), at different times.  Table 2 contains the formulation of the mouthwash test. In the present study, a Quigley-Hein plaque index was used. The patients were submitted to a (preclinical) period of 24 hours without the use of oral care products and their plaque index (PI) was measured. For this analysis, basic fuchsin was used in the form of two tablets that were chewed and spread over all dental surfaces in order to establish the baseline index where the stained surfaces were marked in an appropriate dental chart [35].

Conventional brushes and toothpaste (soft brush and low abrasive dentifrice and no active ingredient) were provided to the twelve individuals who brushed their teeth and rinsed with the rinses formulations for 1 minute. New plaque disclosing and new dental chart with marked plaque index were performed.

Brushing and mouthwash procedures mentioned were made four times a day for a period of a week when new disclosure and new scores were marked. Before the replacement of mouthwash with another, there was another washout period in which the patients remained for a similar period of 24 hours without taking any kind of oral hygiene; to establish a new database and a new study of a formulation by a period of one week up to 4 groups used 4 formulations rinses. Thus, each experimental mouthwash was tested for a week in each subject. The total elapsed time during the full clinical study was 4 months.

A computer program generated randomization groups. The four products were compared between themselves. Also, the three different times for each product were compared too. A nonparametric Friedman test was used for these comparisons. When the Friedman test showed significant differences, Wilcoxon test for disclosure of different pairs to each other was performed. The program used for statistical analysis was SPSS for Windows, version 17 (SPSS, Chicago, IL, USA). For all tests, those differences were considered significant, where *P* < 0.05.

## 3. Results

**Figure 1:**
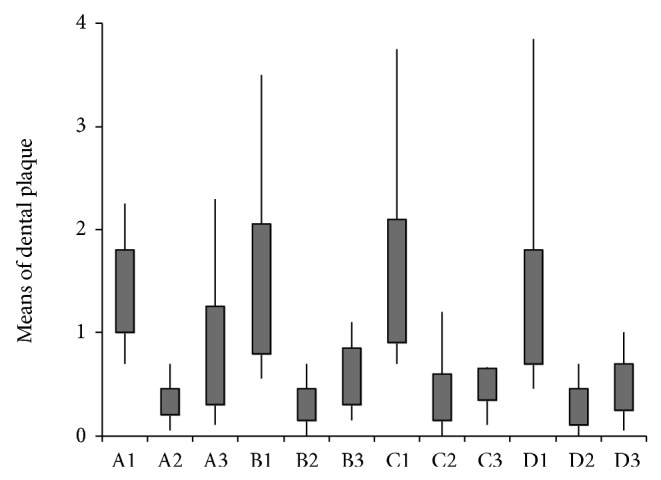
Mean values of biofilm for four solutions (A: Control; B: Plax; C:* B. dracunculifolia* extract and essential oil; D: Listerine) in three different experimental times (1: preliminary: basal level; 2: immediately after: measured after one-time use of mouthwash; 3: one week: a full week of mouthwash use).

Figure 1 illustrates the results of the plaque index (biofilm) recorded at each time for each mouthwash solution. The PI was measured by scores (ordinal categorical variable). Three rinses and one control Three rinses and one control (Listerine, Plax, formulation/testing with active component and basic formulation without active component) in 3 days (basal level, immediately after first use, and after one week of use) were tested to verify the modification of supragingival biofilm found in these times, with each of these solutions.

The comparison between the products in each time with the preliminary time (washout 24 hours, *t* = 0) does not present difference in plaque index (PI) of individuals for all products evaluated (*P* = 0.510), so individuals started the study with a similar index.

In measuring the time after use of mouthwash (*t* = 1), there was no difference between products (*P* = 0.602); that is, subjects in the study had a similar PI after the first use. After one week (*t* = 2), there was no difference between the four evaluated products (*P* = 0.674); that is, all research individuals completed the study with a similar reduction in plaque between themselves and different from initial state. To check which rinses differed during the different times (*P* < 0.05 in the Friedman test), each mouthwash was compared using the Wilcoxon test. Significant differences were observed (*P* < 0.05) in pairs, as we have shown: (A) there was significant difference between times (Friedman, *P* < 0.001); (B) there was no significant difference between the times (Friedman, *P* < 0.001); (C) there was no significant difference between the times (Friedman, *P* < 0.001); (D) there was significant difference between times (Friedman, *P* < 0.001).

## 4. Discussion

Propolis is a honeybee product that has been studied worldwide. It has been considered as food and nutraceutical products because of its medicinal characteristics [22]. Propolis contains a wide range of biological attributes, including antioxidant, antibacterial, antifungal, antiviral, antitumor, anti-inflammatory, and hepatoprotective activities [36, 37]. Such effects have been attributed to the presence of polyphenols, such as flavonoids and phenolic acids, in its composition [38].

*B. dracunculifolia* is used by bees to produce Brazilian green propolis. Besides,* B. dracunculifolia* is a medicinal plant used to prevent and treat diseases [39]. In the present study, the capability of* B. dracunculifolia* that decreases dental biofilm to protect against dental caries was investigated.

Our results revealed that the test formulation with active* B. dracunculifolia* reduced the rate of plaque (biofilm) after one week of use, Our results revealed that the test formulation with active* B. dracunculifolia* reduced the rate of plaque (biofilm) after one week of use, in the same level as chloride triclosan, Gantrez, and essential oils, that are established products in market used as agents of control plaque and halitosis of oral origin [40].

About the relation between* B. dracunculifolia* and oral diseases, there are only a few studies in the literature discussing a possible therapeutic application of these extracts in maintaining oral health. Leitão et al. [30] determined and compared the effects of green propolis and* B. dracunculifolia* extracts on the acid production and synthesis of glucans by glucosyltransferases from* S. mutans*. They investigated biological activities of secondary metabolites of* B. dracunculifolia* on cariogenic factors of* S. mutans* and corroborated the present study, since both suggest that* B. dracunculifolia* extracts could be successfully incorporated into pharmaceutical products employed in dental care. Also, we compared this plant with market products already used as agents for control of plaque and halitosis.

The inhibition of glucosyltransferase (GTF) activity is one of the mechanisms proposed to explain the action of* B. dracunculifolia* extract for caries prevention [30]. GTFs are important factors in the inhibition of bacterial cellular adherence in caries prevention. The glucans synthesized by GTFs promote the accumulation of cariogenic* Streptococci* on the tooth surface and contribute significantly to the production and development of dental plaque [41]. In this way, experimental data suggests that extract of* B. dracunculifolia* may inhibit bacterial metabolic reactions that precede cell doubling or may trigger precocious bacterial cell multiplication as an answer to the aggressive agent [30].

The production of acids from fermentable carbohydrates is one of the cariogenic factors of* S. mutans* that receive attention during the investigation of new medicines to be applied in dental care. In this way, the reduction effect of extract of* B. dracunculifolia* on the acidogenic potential of* S. mutans* is known, since it decreases bacterial acid production [30].

Essential oil of* Baccharis dracunculifolia* has been studied because of its exotic, strong, and long lasting aroma, which is highly prized by the perfume industry [42]. In the present clinical study, this remarkable feature was clearly evidenced by the reports of the patients who referred to the pleasant flavor and striking long-lasting taste during use and even the after taste. Preliminary study of sensory perception was performed in Preliminary study of sensory perception was performed in patients by a pharmaceutical industry. This work was conducted prior to this trial, so we can choose from four final formulations to produce the best market features. This excellent acceptance of volunteers for taste, quality foam, pH balance, color, and volume is a strong indicator of the attractiveness of the product to market rinses, which tends towards acceptance of natural products.

This study sought to maintain the liquid phase, color stability, aroma, and taste during the study period (4 months) with suitable foaming and no undesirable side effects during treatment. Among various systems used as carriers and with the release of active substances systems, the microemulsions can be highlighted, that is, emulsions formed by droplets smaller than 1 *μ*m, which are presented as net, transparent, and thermodynamically stable isotropic dispersions consisting of water, oil, and surfactants [43]. Commonly, they exhibit various biological and pharmaceutical interesting properties, including biodegradability, biocompatibility, physical stability, and the ease of obtaining. Literature data demonstrate that the emulsions have long been used as carriers for lipophilic drugs [44], in stabilizing substances susceptible to hydrolysis, and in reduction of irritation or toxicity of some drugs [45]. These characteristics can explain the great acceptance of emulsion in present work.

*B. dracunculifolia* mouthwash is alcohol-free and, unlike those of chlorhexidine base, shows no incompatibility with previous use of toothpastes. There were no complaints of burning sensation after using this herbal product, unlike essential oils, whose assets are responsible for the sensation of burning mouth after use. It is known that the use of chlorhexidine before 30 seconds after toothbrushing is incompatible with toothpaste containing amphoteric detergents and sodium monofluorophosphate [46]. On the other hand, the chlorhexidine effectiveness has been higher than the use of triclosan [47]. However, due to chlorhexidine disadvantages, it is necessary to search for ideal antiplaque agent, which is not available [48]. In order to solve this problem, the demand for natural products has been considered. Medicinal plants have been widely used to treat a variety of infectious and noninfectious diseases, and 25% of the commonly used medicines contain compounds isolated from plants [49].

Despite the limitations of this study as sample size, study period, and the subjects' health conditions, this is the first work to report the comparison between a natural product and established commercial products as mouth rinses application. Based on the result that there is the same efficiency of the* B. dracunculifolia* and already marketed mouthwashes, we suggest the use of this natural substrate for prevention and reduction of dental biofilm, as well as caries disease, and more investigations are needed in order to explore the potential characteristics of* B. dracunculifolia* as a specific oral antimicrobial.

## 5. Conclusions

Collectively, our findings demonstrate there was no significant difference between different products used in this study, proving the effectiveness of hygiene complemented by mouth rinses in reducing the biofilm and the good effect of* B. dracunculifolia* in oral treatment. The importance of this study is due to the natural characteristics of the new material that leads to advantages not found in existing products; however, this study should be completed with more investigations and studies, to explore the product in long term follow-up and laboratory tests to improve all the effects and side effects of the new product, since it will be used as medical product.

## Figures and Tables

**Table 1:** Summary of the products tested for efficacy in controlling biofilm.

Group	Product	Fabricant	Active ingredient
A	Basic formulation without active component	FCFRP-USP	Control
B	Plax	Colgate Palmolive, a company	Triclosan + Gantrez + NaF + alcohol (7.5%)
C	Formulation and testing with active component	FCFRP-USP	Extract and essential oil of *B*. *dracunculifolia *
D	Listerine	Johnson & Johnson	Essential oil + alcohol (23.6%)

FCFRP-USP: Faculty of Pharmaceutical Sciences of Ribeirão Preto of University of São Paulo, Brazil.

**Table 2:** Composition of **C** formulation—mouthwash with *B. dracunculifolia*.

Ingredients	Function	(% *p*/*p*)
Sodium fluoride	Active	0.05
Sodium benzoate	Conservative	0.10
Sodium saccharin	Sweetener	0.15
Xylitol	Sweetener	2.50
Menthol	Freshness	0.20
Methylparaben	Conservative	0.10
Mint flavor	Corrective	0.20
Essential oil of *B. dracunculifolia *	Active/oil phase	0.04
Hydroethanolic extract of *B. dracunculifolia *	Main active component	0.16
PEG 40	Surfactant	6.59
Sorbitol	Cosurfactant	6.59
Glycerol	Cosurfactant	6.59
Purified water	Vehicle	100.00
